# Multidimensional proximities and interorganizational coinnovation performance: The roles of intraorganizational collaboration network inefficiency

**DOI:** 10.3389/fpsyg.2023.1121908

**Published:** 2023-02-16

**Authors:** Jie Xu, Chongfeng Wang, Yunzhou Cui

**Affiliations:** ^1^School of Economics, Ocean University of China, Qingdao, China; ^2^School of Business, Qingdao University, Qingdao, China

**Keywords:** multidimensional proximities, interorganizational coinnovation performance, intraorganizational collaboration network inefficiency, QAP model, 5G patent data from China

## Abstract

In a gradually more interlinked world, the formation of collaborations with partners is increasingly regarded as an important driver for generating innovation. Although multidimensional proximities are important factors influencing interorganizational coinnovation performance, relevant empirical studies have not reached consistent conclusions. By focusing on organizational dyad and including intraorganizational collaboration network inefficiency as a moderating variable, we explore the effects of multidimensional proximities on interorganizational coinnovation performance. By reference to 5G patent data collected in China between 2011 and 2020, the research results based on the quadratic assignment procedure (QAP) model show that geographical proximity, cognitive proximity, and institutional proximity all improve interorganizational coinnovation performance. In addition, the inefficiency of intraorganizational collaboration networks decreases the positive effect of geographical proximity but increases the positive effects of cognitive and institutional proximity in this context. These findings have both theoretical and practical implications for organizational partner selection.

## 1. Introduction

As environmental uncertainty, knowledge specialization and dispersion, and task complexity increase, organizations find it increasingly difficult to develop the large variety of complementary knowledge needed to innovate effectively in-house (Capaldo and Petruzzelli, 2014). At the same time, as the body of knowledge needed for innovation purposes becomes more divisible, the ensuing ‘changing technology of technological change’ allows for an increasing division of innovative labor among large numbers of actors (Arora and Gambardella, 1994). Thus, organizations resort to ‘open’ strategies, which allow the outflow and inflow of information across organizational boundaries (Chesbrough, 2003). In a gradually more interlinked world, the formation of collaborations with partners is increasingly regarded as an important driver for generating innovation (Huggins et al., 2012; Guan and Liu, 2016).

In this paper, we try to illuminate how multidimensional proximities as important characteristics affect interorganizational coinnovation performance by focusing on organizational dyad. The multidimensional proximities of the collaboration partners are important factors influencing coinnovation performance (Broekel and Boschma, 2012; Capaldo and Petruzzelli, 2014). Although how geographical proximity shapes interorganizational coinnovation performance has been a classic subject in economic geography, recent research has emphasized alternative types of proximity, such as social, organizational, institutional and cognitive (Boschma, 2005). Nonetheless, empirical studies have not yet reached a consistent conclusion regarding the impacts of multidimensional proximities (Broekel and Boschma, 2012; Cassi and Plunket, 2014). Boschma and Frenken (2011) refer to the differential effects of multidimensional proximities as the ‘proximity paradox’. The existence of the ‘proximity paradox’ indicates that the mechanism associated with the process by which multidimensional proximities affect interorganizational coinnovation performance must be explored in further detail.

To resolve this ‘proximity paradox’, in this paper we shift the focus of our research from the organizational ego to organizational dyad. Most previous studies have measured the multidimensional proximities of organizations in terms of the average degree of proximities between organizations and their partners and explored the impacts of these attributes on organizational innovation (Weterings and Boschma, 2009; Broekel and Boschma, 2012; Presutti et al., 2019). In addition, although some studies have explored the impact of multidimensional proximities from the perspective of organizational dyad, these studies have mainly focused on dynamic evolution, that is, the impacts of multidimensional proximities on the formation of collaborations (Balland, 2012; Cassi and Plunket, 2015; Hansen, 2015; Fitjar et al., 2016), rather than the impacts of multidimensional proximities on interorganizational coinnovation performance (Capaldo and Petruzzelli, 2014; Cassi and Plunket, 2014; Nan et al., 2018). Unlike a focus on organizational ego, a focus on organizational dyad does not average the multidimensional proximities between the organizations and their partners but rather considers the multidimensional proximities of the organizations and their various partners in a more detailed way, which calls for high research precision. Furthermore, according to Boschma’s theory of proximity (Boschma, 2005), shortcomings of multidimensional proximities such as the ‘lock-in’ effect may primarily occur in contexts in which organizations are embedded but not in specific dyad. When focusing on dyad, multidimensional proximities are mainly conducive to improving the amount of information that organizations can obtain and the speed the process obtained information. These two factors which emphasized by Mindsponge theory can further benefit interorganizational coinnovation performance (Vuong and Napier, 2015; Vuong et al., 2022a,b; Vuong, 2023). This means that the impacts of multidimensional proximities may depend on the considered perspectives.

In addition, we also try to explore the moderating roles of intraorganizational collaboration network inefficiency in the process by which multidimensional proximities affect interorganizational coinnovation performance. In fact, multidimensional proximities emphasize only interorganizational collaborations. In addition to interorganizational collaborations, there are also many collaborations between inventors within organizations. The structures of intraorganizational collaboration networks may moderate the influences of multidimensional proximities on coinnovation performance. According to social network theory, information transmission and knowledge heterogeneity of organizations are both affected by the structures of their intraorganizational collaboration networks (Yayavaram and Ahuja, 2008; Wang et al., 2014; Guan and Liu, 2016). Inefficiency as a main network structure indicates low connectivity and diffuse information slowly (Lazer and Friedman, 2007; Fang et al., 2010). However, it also stimulates and preserves knowledge heterogeneity due to collaborations out of discipline which emphasized by 3D multiple filters in Mindsponge theory (Funk, 2014; Vuong and Napier, 2015; Vuong et al., 2022a,b; Vuong, 2023). Geographical proximity boosts interorganizational information sharing but requires efficient intraorganizational networks to deal with the large amount of information obtained externally. Cognitive proximity and institutional proximity help organizations better understand their partners which have similar knowledge bases and routines, increase the information process speed, but need inefficient intraorganizational networks to maintain knowledge heterogeneity. Therefore, intraorganizational collaboration network inefficiency may also be an important factor for exploring coinnovation performance. The exploration of the collaboration networks within organizations can help us better understand the condition by which multidimensional proximities impact interorganizational coinnovation performance.

In summary, based on patent data concerning the 5G (fifth-generation mobile communication technology) field in China from 2011 to 2020, by focusing on organizational dyad and including intraorganizational collaboration network inefficiency as a moderating variable, we explore the effects of multidimensional proximities on interorganizational coinnovation performance. Because collaborations between organizations maintain certain organizational and social proximity, we mainly focus on the effects of geographical proximity, cognitive proximity, and institutional proximity in this paper. Such relational data are characterized by frequent row/column/block autocorrelation and therefore standard tools of inference are problematic (Broekel and Boschma, 2012; Guan and Yan, 2016), therefore we use the QAP model which has been widely used for relational data to estimate our empirical models. We find that all three multidimensional proximities improve interorganizational coinnovation performance. By adding interaction terms of intraorganizational collaboration network inefficiency and multidimensional proximities in QAP models, we find that the inefficiency of intraorganizational collaboration networks decreases the positive effect of geographical proximity but increases the positive effects of cognitive proximity and institutional proximity.

We take the 5G field as our research object for the following reasons. First, technological innovation in the 5G field is more difficult and requires more collaborations than innovation in other fields. Hence, collaborations among organizations and inventors within organizations are more extensive in this field. Second, the ‘patent jungle’ phenomenon has emerged in the context of technological innovation competition, and a large amount of available patent data is conducive to coinnovation research in the 5G field.

The theoretical contributions of this paper are mainly reflected in the following two aspects. First, we shift the focus from the organizational ego to organizational dyad which calls for high research precision and provides more empirical evidence regarding the effects of multidimensional proximities on interorganizational coinnovation performance. Moreover, we argue that the shortcomings of multidimensional proximities such as the ‘lock-in’ effect may primarily occur in contexts in which organizations are embedded but not in specific dyad. This means that the impacts of multidimensional proximities may depend on the considered perspectives. This can be used to explain the ‘proximity paradox’. Second, based on social network theory, intraorganizational collaboration network inefficiency, multidimensional proximities and interorganizational coinnovation performance are situated within a single framework. Multidimensional proximities emphasize only interorganizational collaborations; however, inefficiency, as a main structural characteristic of intraorganizational collaboration networks, moderates the process by which multidimensional proximities affect interorganizational coinnovation performance. Inefficiency indicates low connectivity and diffuse information slowly, however, it also stimulates and preserves knowledge heterogeneity. When exploring the effects of multidimensional proximities, we should further consider the structures of intraorganizational collaboration networks. Based on our results, we recommend that, in the case of coinnovation, organizations should collaborate with partners with high levels of geographical proximity, cognitive proximity and institutional proximity to achieve high coinnovation performance. However, different partner selection strategies need different intraorganizational network structures. When selecting partners with geographical proximity, organizations should keep their intraorganizational collaboration networks efficient; however, when selecting partners with cognitive proximity or institutional proximity, they should construct inefficient intraorganizational collaboration networks.

The paper is organized as follows: Section 2 drives theory of innovation and information process, and our hypotheses regarding the effects of multidimensional proximities on interorganizational coinnovation performance. Section 3 provides a description of the data and the method of network construction and describes the estimation design. Section 4 presents the results and Section 5 provides the discussion, conclusions, practical implications, and limitations of our analysis.

## 2. Theory and hypotheses

### 2.1. Innovation and information process

The first definition of innovation was coined by Schumpeter in the late 1920s (Hansen and Wakonen, 1997), who stressed the novelty aspect. According to Schumpeter, innovation is reflected in novel outputs: a new good or a new quality of a good; a new method of production; a new market; a new source of supply; or a new organizational structure, which can be summarized as ‘doing things differently’ (Crossan and Apaydin, 2010). Although Schumpeter clearly positioned his definition of innovation within the domain of the firm and outlined its extent as product, process, and business model, there are continuing debates over various aspects of innovation. According to Crossan and Apaydin (2010), innovation can be seen as a process which has six dimensions: level, drive, direction, source, locus and nature; or innovation can be seen as an outcome which also has five dimensions: form, magnitude, referent, type and nature. In summary, it is hard to describe innovation (Vuong and Napier, 2014). Besides, there are also some scholars focus on the concept of creativity. Although creativity scholars have primarily underlined the importance of generation, or coming up with a novel and useful idea, innovation scholars have stressed the importance of the implementation of the idea and its effects on the field (Perry-Smith and Mannucci, 2017), sometimes they are often interchangeable (Vuong and Napier, 2014). In this paper, we also take the view that they are interchangeable and focus mainly on the technological innovation based on organizational dyad level.

According to Mindsponge theory, the input for generating innovation is information. Useful insights are the outcomes of the information filter after evaluating, connecting, comparing, and imagining based on information input. The Mindsponge framework delves deeper into the mechanism of how the information is learned and unlearned through a constantly updating multi-filtering system. There are two major filters in multi-filtering system, which are 3D multiple filters and trust evaluator. Therefor, to increase the probability of generating innovation, organizations have to increase the number of useful insights, which can be achieved by increasing the amount of information and the processing speed (Vuong and Napier, 2015; Vuong et al., 2022a,b; Vuong, 2023). The above two determinations of innovation also apply to interorganizational coinnovation. During collaboration, organizations learn from each other to achieve high coinnovation performance. The more information they receive and the faster they process information, the more they can learn from each other. In the following hypotheses related to multidimensional proximities, we will illustrate how they affect coinnovation performance through their relations with the amount of information and the speed of information processing. In the hypothesis related to the inefficiency of intraorganizational collaboration networks, we will illustrate how it moderates the effects of multidimensional proximities through its relations with information processing speed and knowledge heterogeneity.

### 2.2. Geographical proximity and coinnovation performance

Geographical proximity is the spatial vicinity of the organizations’ physical locations (Balland et al., 2015). Geographical concentration is an important feature of many industries (Sorenson and Audia, 2000). Such proximity offers benefits such as lower transportation costs and convenient access to skilled labor (Porter and Stern, 2001). Regarding innovation, however, often the greatest advantages of being located near other organizations are those resulting from increased access to information (Funk, 2014). Geographical proximity, as a coordination mechanism (Cassi and Plunket, 2014), will facilitates information sharing between organizations, thus helping organizations obtain a large amount of external information (Vuong and Napier, 2015; Vuong et al., 2022a,b; Vuong, 2023). Specifically for the following reasons. First, geographical proximity allows an increased chance for face-to-face interaction, thus facilitating the timely exchange of information and ideas and the mutual understanding of both parties’ technologies (Malmberg and Maskell, 2006; Nan et al., 2018). Second, geographical proximity is conducive to the formation of social ties (Nan et al., 2018) and increases the probability of individuals within organizations attending the same meetings, in which a great deal of information can be shared among organizations. Third, repeated contact among geographically proximate organizations gradually fosters the formation of collaborative routines, thus reducing the hazard and risk associated with collaborations and increasing the willingness and motivation of both parties to share information (Galunic and Rodan, 1998). Interorganizational coinnovation and coinnovation performance can be enhanced by using such external information (Dongling et al., 2022). Hence, we propose the following hypothesis:

*H1*: Geographical proximity positively affects interorganizational coinnovation performance.

### 2.3. Cognitive proximity and coinnovation performance

Cognitive proximity is the extent to which two organizations share the same knowledge base (Nooteboom, 1999; Balland et al., 2015). Organizations require a certain common knowledge base to process the information obtained from each other. Without a common knowledge base, even if an organization can obtain the information possessed by other organizations, it is difficult for the first organization to internalize, adapt and use that information (Boschma, 2005; Funk, 2014). In Mindsponge theory, there are two mechanisms that can be used to process information, the first one is 3D multiple filters, which emphasizes the important roles of expertise within discipline, collaborations out of discipline and disciplined process (Vuong et al., 2022a,b; Vuong, 2023). Among the 3D multiple filters, expertise within discipline is ensured by the cognitive proximity both between organizations and within organizations. Specifically, cognitive proximity, as a learning mechanism (Cassi and Plunket, 2014), is beneficial to interorganizational coinnovation performance for the following reasons. First, organizations need heterogeneous knowledge for innovation, but the task of acquiring and integrating heterogeneous knowledge is difficult. A certain common knowledge base is necessary to discover opportunities for technical collaborations among organizations (Jaffe, 1986; Cohen and Levinthal, 1990; Cantner and Meder, 2007). Similar knowledge bases entailed by cognitive proximity facilitate the process of absorbing information, thereby enhancing the efficiency of collaborations. Second, cognitive proximity enhances the quality of communication among organizations. The more similar the organizations’ knowledge bases are, the fewer obstacles they face in the communication process, which in turn decreases communication costs and increases the possibility of coinnovation (Callois, 2008). Therefore, cognitive proximity facilitates the internalization, adaptation and use of information (Guan and Yan, 2016), thus benefitting interorganizational coinnovation performance. Cognitive proximity may also increase the risk of involuntary spillovers; in such circumstances, competitors are very reluctant to share information (Boschma, 2005). However, collaborations between organizations are not unconscious but rather the choice of organizations. Rather than involuntary spillovers, collaborations mainly reflect voluntary knowledge transfer. In addition, collaborations between organizations maintain certain organizational and social proximity, which can also be used to weaken opportunistic behaviors (Boschma, 2005). Accordingly, we propose the following hypothesis:

*H2*: Cognitive proximity positively affects interorganizational coinnovation performance.

### 2.4. Institutional proximity and coinnovation performance

Institutional proximity is high when actors operate under the same set of norms and incentives (Balland et al., 2015), e.g., when co-located in the same country (Gertler, 1995; Hoekman et al., 2009) or operating in the same social subsystem, particularly within academia, industry or government (Etzkowitz and Leydesdorff, 2000; Ponds et al., 2007). We divide organizations into four categories: universities or colleges, companies, research institutes or research centers, and other organizations that do not belong to any of the other three categories. When two organizations are of the same type, they exhibit institutional proximity. In Mindsponge theory, the second mechanism that can be used to process information is trust evaluator (Vuong et al., 2022a,b; Vuong, 2023). Specifically, institutional proximity, also as a learning mechanism (Cassi and Plunket, 2014), is beneficial to interorganizational coinnovation performance for the following reasons. First, organizations with high institutional proximity have similar institutional arrangements and codes of conduct, which makes it easier for them to form trust relationships and reduces the communication and transaction costs resulting from uncertainty, which helps such organizations understand each other (Cassi and Plunket, 2015). Second, organizations with high institutional proximity are more likely to communicate and learn. Due to the positive externality associated with knowledge, mutual learning among these organizations is more frequent (Guan and Yan, 2016). Accordingly, we propose the following hypotheses:

*H3*: Institutional proximity positively affects interorganizational coinnovation performance.

### 2.5. The moderating effects of collaboration network inefficiency within organizations

The collaboration networks within organizations are complex networks formed by inventors. According to social network theory, information transmission and knowledge heterogeneity of organizations are both affected by the structures of their intraorganizational collaboration networks (Yayavaram and Ahuja, 2008; Wang et al., 2014; Guan and Liu, 2016). The main types of network structures include centrality, structural holes, cohesion, and inefficiency. Centrality reflects the quantity of connections belonging to the focal node (Borgatti, 2005), while structural holes reflect the level of disconnection among the nodes connected to the focal node (Burt, 2004), which are both microlevel structures. Cohesion reflects the quantity of redundant ties in the network, while inefficiency reflects the quantity of brokers in the network, which are both macrolevel structures (Funk, 2014). In intraorganizational collaboration networks, cohesion means that most inventors can connect with each other through both direct and indirect collaboration, while inefficiency means that most inventors can connect with each other only through indirect collaboration (through brokers). These two macro structures reflect the same feature from two opposite points of view. Thus, the following hypotheses are mainly focused on inefficiency. In addition, because the research objects of this paper are organizations, the two microlevel structures that focus on inventors in organizations are excluded.

The inefficiency of the intraorganizational collaboration network depends on the quantity of intermediary inventors within the organizations in question (Funk, 2014). First, high inefficiency entails that intraorganizational collaboration networks feature few connections among inventors, so the speed of information transmission is low. Slower information transmission often leads to greater communication costs with regard to interorganizational collaborations. Second, high inefficiency indicates that organizations contain many intermediary inventors who are often able to combine knowledge in novel ways (Burt, 2004). Previous research has claimed that an inefficient intraorganizational collaboration network can enable inventors to generate creative ideas, thus reducing the collaboration of repetitive knowledge within the organization. For example, Lazer and Friedman argue that inefficient networks transmit information less efficiently but support the idea that this kind of network maintains heterogeneity (Lazer and Friedman, 2007). Inefficient intraorganizational collaboration networks create new knowledge more easily (Funk, 2014). 3D multiple filters also highlight the important role of collaborations out of discipline in ensuring knowledge heterogeneity (Vuong et al., 2022a,b; Vuong, 2023).

As discussed in hypotheses 1, 2 and 3, geographic proximity, cognitive proximity and institutional proximity play different roles in interorganizational collaborations; geographic proximity mainly acts as a coordination mechanism that benefits information sharing which help organizations to obtain amount of information; and cognitive proximity and institutional proximity mainly act as learning mechanisms that benefit information understanding which help organizations to increase information process speed (Cassi and Plunket, 2014). As a result, high geographical proximity among organizations may lead to information overload and need organizations to construct more efficient intraorganizational collaboration networks to speed up the information dissemination. This means that the inefficiency of intraorganizational collaboration networks will decrease the positive effect of geographical proximity on interorganizational coinnovation performance. Regarding cognitive proximity and institutional proximity, due to the similarity of knowledge bases and norms, organizations that exhibit cognitive proximity and institutional proximity are more likely to understand information from each other and can thus process it quickly. In this situation, the stronger information processing ability and higher knowledge transmission efficiency caused by the efficiency of intraorganizational collaboration networks intensify the homogenization of organizational ideas (Perry-Smith and Shalley, 2003; Granovetter, 2005; Funk, 2014), which may further impede collaboration. Organizations need an essential inefficient collaboration network to maintain their knowledge heterogeneity (Ahuja, 2000). This means that the inefficiency of intraorganizational collaboration networks will increase the positive effects of cognitive proximity and institutional proximity on interorganizational coinnovation performance. Therefore, we propose the following hypotheses:

*H4*: The inefficiency of intraorganizational collaboration networks decreases the positive effect of geographical proximity on interorganizational coinnovation performance.

*H5*: The inefficiency of intraorganizational collaboration networks increases the positive effect of cognitive proximity on interorganizational collaboration performance.

*H6*: The inefficiency of intraorganizational collaboration networks increases the positive effect of institutional proximity on interorganizational coinnovation performance.

**Figure 1:**
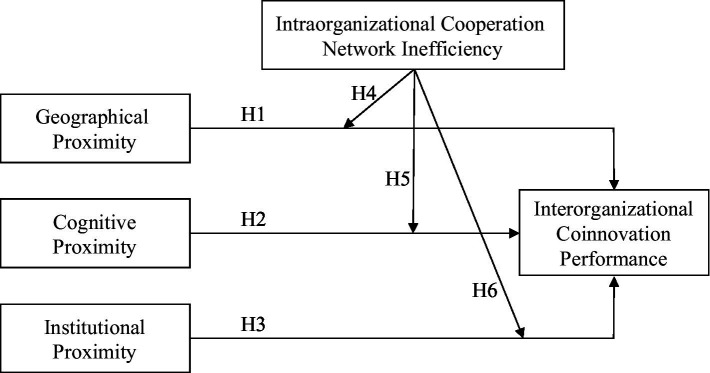
Research framework.

In summary, the research framework of this paper is shown in Figure 1.

## 3. Data and methods

### 3.1. Data

The research object of this paper is the field of 5G technology. Our reasons for choosing this field are as follows. First, technological innovation in the 5G field is more difficult and requires more collaborations than innovation in other fields. Hence, collaborations among organizations and inventors within organizations are more extensive in this field. Second, the “patent jungle” phenomenon has emerged in the context of technological innovation competition, and a large amount of available patent data is conducive to coinnovation research in the 5G field. Patents are widely used to measure the innovation of organizations (Wang et al., 2014; Guan and Liu, 2016). Although patent data have various limitations, for example, they cannot fully represent the innovation capability of organizations and cannot be used to represent organizations’ tacit innovations. However, patent data also offer many advantages, such as objectivity, availability, and quantifiability. Therefore, patent data remain an important source to which academics can refer to measure technological innovation. Accordingly, this paper focuses on the patent data available from the patent information service platform (searc.cnipr.com), and the specific method of data processing used in this paper is as follows:

The first step in this process was to identify patent data related to the 5G field. First, common methods of identifying patent data include IPC (international patent classification) and keyword searches. IPCs can achieve greater clarity with regard to the technical fields involved in the patents than keyword searches (Guan and Liu, 2016). Therefore, we limited the IPC classification numbers involved in the 5G field to H04B, H04H, H04J, H04L, HO1K, H04M, H04Q, H04R, H04N, H04S, and H04W (Yang and Yang, 2019). We searched for patent data available from the patent information service platform (searc.cnipr.com) in September 2020. Subsequently, patent data were divided into utility patents and invention patents. Utility patents emphasize practical value and have lower requirements with regard to creativity and technical ability, while invention patents are more representative of technological innovation capabilities (Stuart and Podolny, 1996); accordingly we took invention patents as our research object. Thereafter, in light of the fact that patent granting lags patent application and is more uncontrollable, the patents granted to an organization during a certain year may not represent the organization’s innovation in that year. Therefore, we searched the patents based on the application date. Ultimately, data regarding a total of 864,483 invention patents were retrieved.

The second step was to clean the patent data. First, samples including incorrect Chinese provincial codes in the patent data were excluded, and only the patent data associated with 31 provincial-level administrative regions in China (excluding Hong Kong, Macao, and Taiwan) were considered. Subsequently, to measure organizational coinnovation performance, we excluded patent data with a single applicant. Finally, we further excluded patents with individual applicants and retained only data concerning patent applications by organizations.

The third step was to match the patent data. After the second step of data cleaning, we obtained 12,329 patent applications from 2011 to 2015 and 20,405 patent applications from 2016 to 2020. First, to avoid the influence of reverse causality and endogeneity, we measured the independent variables based on patent data from 2011 to 2015 and the dependent variables based on patent data from 2016 to 2020 (Singh et al., 2016). Second, to match the earlier samples with the later samples, we selected 1801 organizations as the regression sample and used Python to construct an 1801∗1801 matrix for each variable.

### 3.2. Construction of interorganizational and intraorganizational collaboration networks

**Figure 2:**
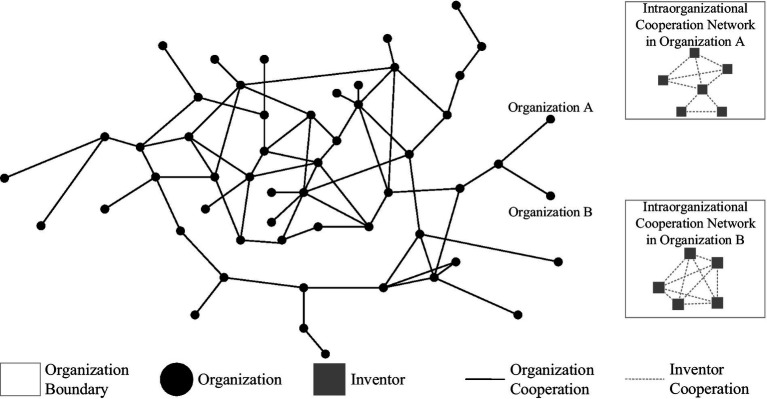
Illustration of interorganizational and intraorganizational collaboration networks.

This article mainly involves the collaboration networks among organizations and the collaboration network within organizations. A patent usually contains multiple applicants (organizations) and multiple inventors. Therefore, we identified interorganizational collaboration networks as collaborations among applicants (organizations) and intraorganizational collaboration networks as collaborations among inventors within organizations. Interorganizational collaboration networks feature organizations as network nodes, and the applicants (organizations) associated with the same patent exhibit a collaboration relationship, thus forming a network connection. Intraorganizational collaboration networks feature inventors as network nodes, and inventors associated with the same patent exhibit a collaboration relationship, which thus constitutes a network connection. The relation between these two kinds of networks is illustrated in Figure 2.

### 3.3. Variable definition

#### 3.3.1. Interorganizational coinnovation performance

Interorganizational coinnovation performance ( CoP ) refers to organizations’ levels of technological innovation in the context of collaborations. In empirical research, due to the time lag and uncontrollability of patent granting, scholars usually use the quantity of patent applications to measure the innovation performance of organizations (Hagedoorn and Cloodt, 2003). Therefore, this paper uses the quantity of patent applications of every two organizations involved in such applications during the period 2016–2020 as a measure of interorganizational coinnovation performance. For patent applications involving more than two organizations, we treat them as coinnovation outcomes for each pair of organizations in these applications. Thus, these applications contribute to the interorganizational coinnovation performance for each pair of organizations in them.

#### 3.3.2. Geographical proximity

Geographical proximity can be calculated by the distance between two organizations in the spatial dimension. First, we used Baidu map software to determine the latitude and longitude of each organization. For organizations whose latitude or longitude could not be determined based on the Baidu map, we used Qichacha, an enterprise checking website, to identify their addresses and confirm the latitude and longitude manually using the Baidu map. Subsequently, referring to (Sampson, 2007) we calculated the geographic proximity ( GP ) between every two organizations as follows:


$$\begin{array}{l} GP_{ij}=-GD_{ij}=r\{\arccos[\sin\left(lat_{i}\right)\sin\left(lat_{j}\right)\\ \;\;\;\;\;\;\;\;\;\;\;\;\;\;\;+\cos\left(lat_{i}\right)\cos\left(lat_{j}\right)\cos\left(\left|long_{i}-long_{j}\right|\right)\} \end{array}$$

where $GP_{ij}$ is the geographical proximity between organization i and organization j , $GD_{ij}$ is the distance between organization i and organization j ; r is the radius of the earth, set at 3963 miles; $lat_{i}$ and $lat_{j}$ represent the latitudes of organization i and organization j , respectively; and $long_{i}$ and $long_{j}$ represent the longitudes of organization i and organization j , respectively.

#### 3.3.3. Cognitive proximity

Cognitive proximity ( CP ) refers to the degree of overlap in technology categories between two organizations (Reuer and Lahiri, 2014). First, we matched the applicants and their patents’ 4-digit IPCs. Second, the types and numbers of IPCs associated with each applicant were counted. Finally, referring to (Guan and Yan, 2016) the cognitive proximity between every two organizations was calculated as follows:


$$CP_{ij}=f_{i}f_{j}^{\prime }/\sqrt{\left(f_{i}f_{i}^{\prime }\right)\left(f_{j}f_{j}^{\prime }\right)}$$

where $CP_{ij}$ is the cognitive proximity between organization i and organization j ; the multidimensional vectors $f_{i}=\left(f_{i}^{1},f_{i}^{2},\ldots ,f_{i}^{N}\right)$ and $f_{j}=\left(f_{j}^{1},\;f_{j}^{2},\ldots ,\;f_{j}^{N}\right)$ can be used to capture the distribution where $f_{i}^{N}$ and $f_{j}^{N}$ indicate the quantity of patents applied for by organization i and organization j in technology classification N , respectively; and apexes indicate transposed vectors. $CP_{ij}$ varies between 0 and 1. When organizations are associated with the same research field, this value equals one.

#### 3.3.4. Institutional proximity

Institutional proximity (IP) is high when actors operate under the same set of norms and incentives (Balland et al., 2015), and we measure such proximity according to the types of organizations (Etzkowitz and Leydesdorff, 2000; Ponds et al., 2007). We divide organizations into four types according to the tail of the applicants’ name: universities or colleges, companies, research institutes or research centers, and other organizations that do not belong to the above three categories. In this paper, dummy variables are set according to whether the sample organizations belong to the same type:


$$IP_{ij}=\{\begin{array}{cc} 1, & organization\;i\;and\;j\;belongtothesametype\\ 0, & organization\;i\;and\;j\;belongtothedifferenttype \end{array}$$

where $IP_{ij}$ is the institutional proximity between organization i and organization j . When the organizations belong to the same type, this value equals one.

#### 3.3.5. Intraorganizational collaboration network inefficiency

Referring to the research of Funk (2014), we selected the average path length of the intraorganizational collaboration network to measure inefficiency. The longer the average path is, the more ‘intermediaries’ exist within the internal collaboration network of the organization. Therefore, we calculate the path length between every pair of inventors who can be connected through limited intermediaries within each organization and subsequently calculate the arithmetic average as follows:


$$IE_{ij}=\frac{\sum_{m,n\in Net_{i}\;and\;m>n}L_{mn}}{N_{i}\left(N_{i}-1\right)/2}+\frac{\sum_{s,t\in Net_{j}\;and\;s>t}L_{st}}{N_{j}\left(N_{j}-1\right)/2}$$

where $IE_{ij}$ is the sum of the intraorganizational collaboration network inefficiency of organization i and organization j ; $m,n\in Net_{i}\;and\;m>n$ and $s,t\in Net_{j}\;and\;s>t$ represent inventors belonging to the intraorganizational collaboration networks of organization i and organization j , respectively; $L_{mn}$ and $L_{st}$ represent the shortest path length between m,n and s,t in the intraorganizational collaboration networks of organization i and organization j , respectively; and $N_{i}$ and $N_{j}$ represent the total quantity of inventors belonging to the intraorganizational collaboration networks of organization i and organization j , respectively. L and N can be calculated in the intraorganizational collaboration networks constructed in Section 3.2 by using UCINET software. L is a network structure that has been widely used in network analysis, when two inventors are connected, the shortest path length between them is equal to 1, when they are not connected but connected the same other inventors, the shortest path length between them is equal to 2, etc.; N represents the quantity of nodes in intraorganizational collaboration networks.

#### 3.3.6. Control variables

Following the suggestions of Guan and Yan (2016), to control for the impact of differences among collaborative organizations and their previous collaboration experience, we introduce patent size ( PS ), number of inventors ( IN ), network structure ( NS ), and coinnovation performance ( PC ) over the last 5 years as control variables. In this context, PS refers to the quantity of patent applications filed by the organizations over the past 5 years (in thousands), which is represented by the difference matrix of the quantity of patent applications between the two organizations from 2011 to 2015 (the values of the difference matrix are all expressed as absolute values); IN is represented by the difference matrix of the quantity of inventors owned by the two organizations from 2011 to 2015 (in thousands); NS is the difference matrix of the structural holes of the organizations in the interorganizational collaboration network; and PC is the matrix of the quantity of collaborative patent applications of the organizations from 2011 to 2015.

### 3.4. Methods

According to Broekel and Boschma (2012) and Guan and Yan (2016), we use the QAP model, which has been widely used in relational data, to estimate our empirical models. Such relational data are characterized by frequent row/column/block autocorrelation, and therefore, standard tools of inference are problematic (Krackhardt, 1987). A solution is the QAP model (Krackhardt, 1988). Compared with OLS regression, the QAP model is superior for testing research hypotheses in models based on relational data (Tsai, 2002). Previous research using Monte Carlo simulations showed that OLS estimates are statistically biased when confronting the autocorrelation problem, yet QAP is relatively unbiased in multiple regressions (Shah, 1998). QAP regresses a dependent matrix on independent matrices through a nonparametric permutation test. It has a permutation regression process that transposes the dependent matrix’s rows and columns and recomputes the regression many times (in this study, 500 times) to estimate the significance. Thus, this method can reasonably help remedy the autocorrelation problem (Tsai, 2002; Lai et al., 2009). Accordingly, we employed the QAP model to conduct correlation analysis and regression analysis using UCINET software.

## 4. Analysis results

### 4.1. Descriptive and correlation statistics

Since the data referenced in this paper were all in an 1801*1801 matrix format, the Pearson correlation coefficients were analyzed using the QAP correlation coefficient module in UCINET. The results regarding the descriptive and correlation statistics are shown in Table 1. First, the means of geographical proximity, cognitive proximity, and institutional proximity are −0.614, 0.459, and 0.526, respectively. The correlation coefficients between geographical proximity, cognitive proximity, institutional proximity and interorganizational coinnovation performance are all positive. These correlation coefficients are consistent with Hypotheses 1, 2 and 3 and are significant at the 1% level. Second, the mean of interorganizational coinnovation performance is only 0.005, and the variance is 0.671, which is much smaller than the mean of patent size, which is 0.106. This finding indicates that individual organizations have many patents but also that the collaborative patents of the sample organizations are relatively small, and the quantity of collaborative patents among organizations varies greatly.

**Table 1:** Descriptive and correlation statistics.

	Mean	Std. dev.	1	2	3	4	5	6	7	8	9
1.CoP	0.005	0.671	1.000								
2.PS	0.106	0.766	0.008**	1.000							
3.IN	0.124	0.547	0.023***	0.881***	1.000						
4.NS	0.359	0.397	−0.002**	−0.020**	−0.028**	1.000					
5.PC	0.002	0.309	0.373***	0.019***	0.058***	−0.002	1.000				
6.GP	−0.614	0.325	0.008***	0.001	0.001	0.000	0.009***	1.000			
7.CP	0.459	0.346	0.011***	0.035***	0.066***	0.009	0.014***	−0.000	1.000		
8.IP	0.526	0.499	0.005***	0.011	−0.022	0.045***	0.003*	0.004***	−0.049***	1.000	
9.IE	3.000	1.311	0.015***	0.342***	0.439***	−0.009	0.029***	0.003***	0.201***	−0.071***	1.000

*, ** and *** indicate statistical significance at the of 10, 5 and 1% levels, respectively.

### 4.2. Quadratic assignment procedure regression analysis

The results of the QAP regression analysis are shown in Table 2. In Table 2, Model 1 is the benchmark model, which considers only control variables. Models 2, 3 and 4 add geographical proximity, cognitive proximity, and institutional proximity, respectively. Model 5 considers all independent variables. Models 6, 7 and 8 further include the interaction terms of intraorganizational collaboration network inefficiency, and each contains one of the three independent variables. Model 9 includes all interaction terms of intraorganizational collaboration network inefficiency.

**Table 2:** Regression results regarding the influence of multidimensional proximities and intraorganizational collaboration network inefficiency based on the QAP model.

	Model 1	Model 2	Model 3	Model 4	Model 5	Model 6	Model 7	Model 8	Model 9
Constant	0.010	0.010	0.010	0.010	0.010	0.010	0.010	0.010	0.010
PS	−0.004*	−0.004*	−0.003*	−0.0005**	−0.004*	−0.003*	−0.0009	−0.005**	−0.003*
IN	0.009*	0.009**	0.007*	0.010**	0.008*	0.006	−0.002*	0.005	0.001
NS	−0.004*	−0.004*	−0.004*	−0.004*	−0.004**	−0.004*	−0.004**	−0.004**	−0.004*
PC	0.851***	0.851***	0.851***	0.851***	0.851***	0.851***	0.851***	0.851***	0.851***
GP		0.016***			0.016***	0.025***			−0.016***
CP			0.016***		0.017***		−0.012***		−0.020***
IP				0.008***	0.009***			−0.017***	−0.016***
GP×IE						−0.003**			0.010***
CP×IE							0.009***		0.012***
IP×IE								0.009***	0.009***
*R* ^2^	0.139	0.139	0.139	0.139	0.139	0.139	0.139	0.139	0.139
Adj.*R*^2^	0.139	0.139	0.139	0.139	0.139	0.139	0.139	0.139	0.139

*, ** and *** indicate statistical significance at the of 10, 5 and 1% levels, respectively.

The results of Models 2, 3 and 4 show that geographical proximity, cognitive proximity, and institutional proximity all have positive and significant effects on organizational coinnovation performance ( β=0.016,0.016,0.008;p<0.01 ). Therefore, Hypotheses 1, 2 and 3 are supported. Models 6, 7 and 8 show that the interaction term of intraorganizational collaboration network inefficiency and geographical proximity is negative and significant ( β=−0.003;p<0.01 ); the interaction terms of intraorganizational collaboration network inefficiency and cognitive proximity and institutional proximity are both positive and significant (β=0.009,;0.009,;p<0.01). To explain the moderating effects of intraorganizational collaboration network inefficiency on the relationship between multidimensional proximities and organizational coinnovation performance more clearly, referring to Van de Vrande (2013), we hold the moderating variable at its mean minus one standard deviation ( IE=3.000−1.311=1.689 ), mean ( IE=3.000 ) and mean plus one standard deviation ( IE=3.000+1.311=4.311 ) to represent medium inefficiency, low inefficiency and high inefficiency, respectively. We subsequently calculate the marginal effects of three independent variables. When IE=1.689 , IE=3.000 and IE=4.311 , the marginal effects of geographical proximity are equal to 0.025−0.003∗1.689=0.020 , 0.025−0.003∗3.000=0.016 and 0.025−0.003∗4.311=0.012 , respectively. This finding indicates that the inefficiency of intraorganizational collaboration networks decreases the positive effect of geographical proximity on interorganizational coinnovation performance, thus supporting Hypothesis 4. When IE=1.689 , IE=3.000 and IE=4.311 , the marginal effects of cognitive proximity are equal to −0.012+0.009∗1.689=0.003 , −0.012+0.009∗3.000=0.015 and −0.012+0.009∗4.311=0.027 , respectively. This finding indicates that the inefficiency of intraorganizational collaboration networks increases the positive effect of cognitive proximity on interorganizational coinnovation performance, thus supporting Hypothesis 5. When IE=1.689 , IE=3.000 and IE=4.311 , the marginal effects of institutional proximity are equal to −0.017+0.009∗1.689=−0.002 , −0.017+0.009∗3.000=0.01 and −0.017+0.009∗4.311=0.022 , respectively. This finding also indicates that the inefficiency of intraorganizational collaboration networks increases the positive effect of institutional proximity on interorganizational coinnovation performance, thus supporting Hypothesis 6.

### 4.3. Robustness tests

The dependent variable in this paper is measured in terms of the quantity of collaborative patent applications between organizations, which takes the form of nonnegative integer data. The use of conventional linear regression in this context may lead to bias in the regression estimates. Therefore, the count data model is generally used for regression testing. We use a Poisson regression model to perform robustness tests (Hu et al., 2017). The results of these robustness tests are shown in Table 3.

**Table 3:** Robustness tests regarding the influence of multidimensional proximities and intraorganizational collaboration network inefficiency.

	Model 1	Model 2	Model 3	Model 4	Model 5	Model 6	Model 7	Model 8
PS	−0.277***	−0.249***	−0.285***	−0.230***	0.0190***	0.0114***	0.0291***	0.0143***
	(0.004)	(0.004)	(0.004)	(0.004)	(0.000)	(0.000)	(0.000)	(0.000)
IN	0.755***	0.691***	0.749***	0.651***	0.624***	0.474***	0.501***	0.432***
	(0.005)	(0.003)	(0.003)	(0.004)	(0.004)	(0.004)	(0.004)	(0.004)
NS	−0.430***	−0.494***	−0.495***	−0.525***	−0.333***	−0.309***	−0.343***	−0.302***
	(0.023)	(0.024)	(0.027)	(0.024)	(0.005)	(0.005)	(0.005)	(0.005)
PC	0.0179***	0.0176***	0.0246***	0.0178***	−0.385***	−0.359***	−0.423***	−0.359***
	(0.000)	(0.000)	(0.000)	(0.000)	(0.024)	(0.026)	(0.025)	(0.026)
GP	2.222***			2.075***	6.533***			3.785***
	(0.027)			(0.026)	(0.052)			(0.061)
CP		4.895***		4.547***		2.125***		2.410***
		(0.048)		(0.045)		(0.054)		(0.060)
IP			1.251***	1.183***			−1.162***	0.970***
			(0.021)	(0.021)			(0.029)	(0.046)
GP×IE					−0.818***			−0.310***
					(0.006)			(0.009)
CP×IE						0.585***		0.408***
						(0.004)		(0.009)
IP×IE							0.607***	0.0733***
							(0.004)	(0.008)
Constant	−4.413***	−8.876***	−6.323***	−8.330***	−3.962***	−8.502***	−6.164***	−7.767***
	(0.016)	(0.042)	(0.020)	(0.043)	(0.0150)	(0.0411)	(0.0195)	(0.044)
Obs	3,243,601	3,243,601	3,243,601	3,243,601	3,243,601	3,243,601	3,243,601	3,243,601
*R* ^2^	0.137	0.182	0.125	0.224	0.180	0.233	0.180	0.283

The brackets are heteroscedasticity robust standard errors. *, ** and *** indicate statistical significance at the of 10, 5 and 1% levels, respectively.

As shown in Table 3, the regression results associated with Models 1, 2 and 3 indicate that the effects of geographical proximity, cognitive proximity and institutional proximity are all significant at the 1% level. The coefficients are 2.222, 4.895, and 1.251, respectively. This finding indicates that the positive effects of geographical proximity, cognitive proximity, and institutional proximity on interorganizational coinnovation performance are robust to a certain extent. The regression results associated with Models 5, 6 and 7 indicate that the interactions of inefficiency and multidimensional proximities are all significant at the 1% level. The coefficients are −0.818, 0.585, and 0.607, respectively. This finding indicates that the moderating effects of intraorganizational network inefficiency are also robust.

## 5. Discussion and conclusion

### 5.1. Discussion

Based on patent data concerning the 5G field in China from 2011 to 2020, by focusing on organizational dyad and including intraorganizational collaboration network inefficiency as a moderating variable, we explore the effects of multidimensional proximities on interorganizational coinnovation performance. This study makes several theoretical contributions, as outlined below.

First, we try to illuminate how multidimensional proximities as important characteristics affect interorganizational coinnovation performance by focusing on organizational dyad. Most previous studies have measured the multidimensional proximities of organizations in terms of the average degree of proximities between organizations and their partners and explored the impacts of these attributes on organizational innovation (Weterings and Boschma, 2009; Broekel and Boschma, 2012; Presutti et al., 2019). Although few studies have focused on organizational dyad, they mainly explored the impacts of multidimensional proximities on the quality of existing coinnovation outcomes rather than the quantity of further coinnovation (Capaldo and Petruzzelli, 2014; Cassi and Plunket, 2014; Nan et al., 2018). These studies all used forward citations as indicators of coinnovation performance. However, innovation must satisfy both conditions of originality and effectiveness (Vuong et al., 2022a,b). Besides, these studies all used a standard tool of inference, which may cause statistical bias confronting the autocorrelation problem. Unlike a focus on organizational ego, we use the QAP model and focus on organizational dyad, which do not average the multidimensional proximities between the organizations and their partners but rather consider the multidimensional proximities of the organizations and their various partners in a more detailed way, which calls for high research precision and provides more evidence about how multidimensional proximities affect interorganizational coinnovation performance.

Second, we also try to explore the moderating role of intraorganizational collaboration network inefficiency in the process by which multidimensional proximities affect interorganizational coinnovation performance. In fact, multidimensional proximities emphasize only interorganizational collaborations. In addition to interorganizational collaborations, there are also many collaborations between inventors within organizations. According to social network theory, information transmission and knowledge heterogeneity of organizations are both affected by the structures of their intraorganizational collaboration networks (Yayavaram and Ahuja, 2008; Wang et al., 2014; Guan and Liu, 2016). Inefficiency as a main network structure indicates low connectivity and diffuse information slowly (Lazer and Friedman, 2007; Fang et al., 2010). However, it also stimulates and preserves knowledge heterogeneity due to collaborations out of discipline which emphasized by 3D multiple filters in Mindsponge theory (Funk, 2014; Vuong and Napier, 2015; Vuong et al., 2022a,b; Vuong, 2023). According to Mindsponge theory, to increase the probability of generating innovation, organizations have to increase the number of useful insights, which can be achieved by increasing the amount of information and the processing speed (Vuong and Napier, 2015; Vuong et al., 2022a,b; Vuong, 2023). Geographical proximity boosts interorganizational information sharing but requires efficient intraorganizational networks to deal with the large amount of information obtained externally. Cognitive proximity and institutional proximity increase information processing speed, help organizations better understand their partners which have similar knowledge bases and routines but need inefficient intraorganizational networks to maintain diversity. Our study broadens the conditions associated with the process by which multidimensional proximities affect interorganizational coinnovation performance.

### 5.2. Conclusion

Based on the QAP model, the main conclusions of this research are as follows: With regard to the direct effect of multidimensional proximity on organizational coinnovation performance, geographical proximity, cognitive proximity, and institutional proximity all have significant positive impacts on organizational coinnovation performance. However, multidimensional proximities play different roles in interorganizational collaboration. While geographic proximity mainly acts as a coordination mechanism that benefits information sharing which help organizations to obtain amount of information external, cognitive proximity and institutional proximity mainly act as learning mechanisms that benefit knowledge understanding which help organizations to increase information process speed.

According to detailed consideration of the interaction of intraorganizational collaboration network inefficiency and multidimensional proximities, the interaction term of geographical proximity and intraorganizational collaboration network inefficiency is negative and significant, while the interaction terms of cognitive proximity, institutional proximity and intraorganizational collaboration network inefficiency are both positive and significant. As the inefficiency of the intraorganizational collaboration network increases, intraorganizational information transmission becomes slower, which weakens the positive effect of geographic proximity. However, such intraorganizational collaboration networks are beneficial for generating and maintaining knowledge heterogeneity, which means that the positive effects of cognitive proximity and institutional proximity can be strengthened.

### 5.3. Practical implications

From the perspective of multidimensional proximities and collaboration networks, we provide relevant suggestions for organizations and the Chinese government. First, organizations should take advantage of the concept of proximity and select favorable partners. When an organization selects a partner for coinnovation, it must consider the varying effects of multidimensional proximities on organizational coinnovation performance. With regard to cost, the organization should actively choose partners located in the same region. In addition, the organization can also choose partners with similar knowledge base or institutional proximate, which can effectively improve interorganizational coinnovation performance.

Second, organizations should actively establish appropriate intraorganizational collaboration network structures to strengthen the positive effect of multidimensional proximities. For example, if an organization prefers partners that are geographically proximate, an efficient intraorganizational collaboration network with redundant ties may be better. However, if an organization prefers partners that are cognitive or institutionally proximate, an inefficient intraorganizational collaboration network with intermediary inventors can further enhance the positive effect of proximities.

Finally, organizations should actively utilize relevant information sharing platforms and implement favorable policies. The factor underlying the influence of proximity in this context is the collaborative innovation behavior in which organizations jointly engage. Therefore, on the one hand, the Chinese government can increase the technical exchange and communication channels among organizations by establishing a technology exchange platform and issuing relevant policies regarding technical collaboration and intellectual property management to ensure that organizations can acquire more heterogeneous knowledge to boost coinnovation performance. On the other hand, the Chinese government should continue to strengthen the integrated and coordinated development of collaboration and encourage and promote technical collaboration among various organizations.

### 5.4. Limitations and directions for future research

This paper explores the impact of multidimensional proximities on organizational coinnovation performance and considers the moderating role of intraorganizational collaboration network inefficiency in this context, thus enriching the research on proximity and innovation performance. Nevertheless, this research faces certain limitations. First, our research is based on 5G field patent data. The task of investigating whether this research can be extended to other fields remains worthwhile. In future research, samples should be expanded to other fields to allow us to determine whether there are any differences in the influence of proximity across different fields and different types of organizations. Second, we highlight only the fact that different types of proximity impact organizational coinnovation performance, and the interactions among different types of proximity may also influence organizational coinnovation performance, a topic that can be explored in future research. Third, our research mainly focuses on the moderating roles of intraorganizational collaboration network inefficiency, and future research should be expanded to other network structures.

## Data availability statement

Publicly available datasets were analyzed in this study. This data can be found at: http://search.cnipr.com/.

## Author contributions

JX: conceptualization, methodology, formal analysis, and writing-original draft preparation. CW: formal analysis, writing-review, editing, and supervision. YC: resources, data curation, validation, and supervision. All authors have read and agreed to the published version of the manuscript.

## Funding

This research was funded by the National Social Science Fund of China (No. 20BGL041).

## Conflict of interest

The authors declare that the research was conducted in the absence of any commercial or financial relationships that could be construed as a potential conflict of interest.

## Publisher’s note

All claims expressed in this article are solely those of the authors and do not necessarily represent those of their affiliated organizations, or those of the publisher, the editors and the reviewers. Any product that may be evaluated in this article, or claim that may be made by its manufacturer, is not guaranteed or endorsed by the publisher.
